# Interplay between correlations and Majorana mode in proximitized quantum dot

**DOI:** 10.1038/s41598-018-33529-1

**Published:** 2018-10-24

**Authors:** G. Górski, J. Barański, I. Weymann, T. Domański

**Affiliations:** 1 Faculty of Mathematics and Natural Sciences, University of Rzeszów, 35-310 Rzeszów, Poland; 2 Polish Air Force Academy, ul. Dywizjonu 303, 08-521 Dęblin, Poland; 3 Faculty of Physics, A. Mickiewicz University, 61-614 Poznań, Poland; 4 Institute of Physics, M. Curie-Skłodowska University, 20-031 Lublin, Poland

## Abstract

We study the low energy spectrum and transport properties of a correlated quantum dot coupled between normal and superconducting reservoirs and additionally hybridized with a topological superconducting nanowire, hosting the Majorana end-modes. In this setup the Majorana quasiparticle leaking into the quantum dot can be confronted simultaneously with the on-dot pairing and correlations. We study this interplay, focusing on the quantum phase transition from the spinless (BCS-type) to the spinful (singly occupied) configuration, where the subgap Kondo effect may arise. Using the selfconsistent perturbative treatment for correlations and the unbiased numerical renormalization group calculations we find that the Majorana mode has either constructive or destructive effect on the low-energy transport behavior of the quantum dot, depending on its spin. This spin-selective influence could be verified by means of the polarized STM spectroscopy.

## Introduction

Recent intensive studies of nanoscopic superconductors focused on quasiparticles, which resemble the Majorana fermions^1–7^ that are identical to their own antiparticles. Such exotic objects can appear at defects^8^ or boundaries of topological superconductors^9,10^ and non-Abelian character make them appealing for quantum computing or novel spintronic devices^11^. Although Majorana quasiparticles have been predicted in various physical setups^12–22^, their experimental realization has been so far evidenced in nanowires proximitized to the bulk *s*-wave superconductors by the ballistic tunneling^23,24^, STM measurements^25–28^ and using lithographic structures^29^. Coalescence of the Andreev (finite-energy) bound states into the Majorana (zero-energy) quasiparticles has been also achieved in hybrid structures, comprising quantum dots (QDs) side-attached to topological superconducting nanowires^30,31^. This phenomenon, initially predicted by E. Vernek *et al*.^32^, has been investigated theoretically by various groups^33–35^ and quantum dots proved to be convenient testing grounds of the Majorana modes.

**Figure 1:**
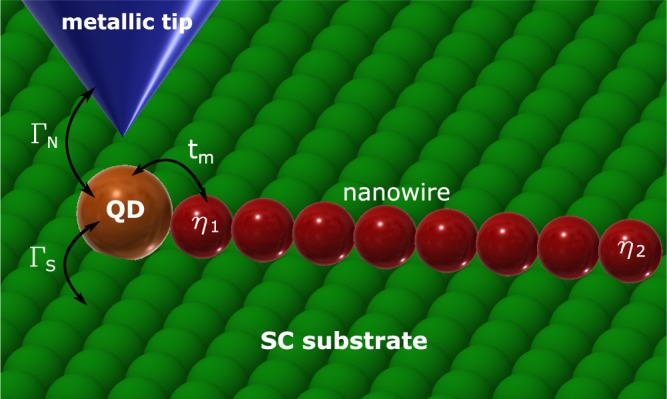
Schematic view. Quantum dot (QD) deposited on superconducting substrate (S) and hybridized with the Rashba nanowire [hosting the Majorana end-modes *η*_1_ and *η*_2_], which is probed by metallic tip (N) via the Andreev tunneling.

Inspired by the high precision scanning-tunneling-microscopy (STM) of hybrid structures^31^ we consider the setup (Fig. 1) in which the leaking Majorana mode is confronted simultaneously with (i) the electron correlations and (ii) the proximity-induced pairing. Correlation effects have been previously addressed on the Hartree-Fock level^36^, using the equation of motion approach^37^ and numerical renormalization group (NRG) technique^38^, but mainly in the weak coupling Γ_*N*_ limit. Our present analysis is complementary to the former studies, focusing on the subgap features (including Kondo effect) of the correlated quantum dot near its changeover between the BCS-type (spinless) and singly-occupied (spinful) configurations^39,40^. Since the Kondo and Majorana features show up at zero-energy, we shall analyze their interplay and check whether they compete, cooperate or have some other relationship. Such interplay could be encountered in STM-type geometries, analogous to what has been reported by the Princeton^25^ and Basel^26^ groups. One can use e.g. nanochains of Fe atoms deposited on superconducting substrate (like Pb or Al) with additional side-coupled adatoms, probing them either by the normal^25,26^ or ferromagnetic^28^ STM tip. In this regard the remarkable technological progress has been recently achieved by H. Kim *et al*.^31^, who fabricated Fe chains (comprising from 3 to 40 atoms) on the surface of superconducting Re using single-atom manipulation method. When attaching individual Fe atoms to the already existing nanochain, the spin-polarized STM measurements (using PtIr tips) inspected emergence of the Majorana modes from the Andreev bound states and controlled ongoing evolution of quasiparticles in the nanochain. Such atom-by-atom construction of nanoscopic hybrids can help verifying the subtle interplay of the Majorana quasiparticles with the subgap Kondo effect. In what follows, we study this issue in a systematic way predicting novel spin-resolved signatures.

## Results

In the absence of Majorana quasiparticles the interplay between correlations and proximity induced on-dot pairing has been investigated in N-QD-S junctions by several groups^39–41^. Here we study the role of Majorana mode, exploring its influence on the subgap electronic states. Our major concern is to focus on the quantum phase transition/crossover from the spinful (singly occupied) to the spinless (BCS-type) configurations^39,40^, when the Andreev bound states cross each other. Analysis of correlations and their relationship with the Majorana quasiparticles has been previously performed mainly for the QD embedded between both metallic^32,42–49^ or ferromagnetic^50,51^ electrodes. Signatures of the leaking Majorana quasiparticle have been predicted in the tunneling conductance. In particular, for the long nanowires (with negligible overlap between the Majorana modes), the linear conductance should reach 3*e*^2^/2*h*, whereas for short ones (with the overlapping Majoranas) its value would approach 2*e*^2^/*h*^43,44,50,51^. Thermoelectric properties of such N-QD-N setup revealed that the thermopower is going to reverse its sign^43,50,51^. For junctions, comprising one normal and another superconducting electrode, the influence of the Majorana quasiparticle on the low-energy (subgap) spectrum of correlated QDs is much less explored^37,38,46,52^. Due to the induced electron pairing^53,54^ any physical process for a given spin would simultaneously affect its opposite counter-partner^55^. To be more specific, the Majorana quasiparticle hybridized with, let us say spin-↑ electron, would also affect the spectrum of the spin-↓ electrons. Here we find that, despite this mixing, the leaking Majorana mode has *spin-selective influence on the subgap quasiparticles*. We discuss this phenomenon for uncorrelated and correlated quantum dots, respectively. Moreover, since both spin components are important for the Andreev scattering processes, we examine in detail the resulting subgap transport properties.

### Low energy microscopic model

Practical realizations of topological superconducting phase in semiconducting wires^23,24^ or magnetic atoms’ chains^25–28^ rely on *p*-wave pairing (of identical spins) between the nearest neighbor sites, reminiscent of the Kitaev toy model^10^. Let us assume that such inter-site pairing is induced between ↑ electrons (we shall revisit this assumption in the last subsection) and only this particular spin component of the QD is *directly* coupled to the Majorana quasiparticle^32,56^. Via the proximity induced on-dot pairing, the other (↓) spin would be *indirectly* affected by the Majorana quasiparticle. Effectively, any process engaging spin-↑ electrons would simultaneously (although with different efficiency) affect the opposite spin^55^. This is important for the particle-to-hole conversion scattering mechanism, contributing to the subgap charge transport at low temperatures.

On a microscopic level, our setup (Fig. 1) can be described by the Anderson-type Hamiltonian

1 $$H=\sum _{\beta =S,N}\,({H}_{\beta }+{H}_{\beta -QD})+{H}_{QD}+{H}_{MQD},$$

where ${H}_{N}={\sum }_{k,\sigma }\,{\xi }_{kN}{c}_{k\sigma N}^{\dagger }{c}_{k\sigma N}$ describes the metallic electrode, ${H}_{S}={\sum }_{k,\sigma }\,{\xi }_{kS}{c}_{k\sigma S}^{\dagger }{c}_{k\sigma S}-{\sum }_{k}\,({\rm{\Delta }}{c}_{k\uparrow S}^{\dagger }{c}_{-k\downarrow S}^{\dagger }+h\mathrm{.}c.)$ refers to *s*-wave superconducting substrate and electron energies *ξ*_*kβ*_ are measured with respect to the chemical potentials *μ*_*β*_. The correlated QD is described by ${H}_{QD}={\sum }_{\sigma }\,\varepsilon {d}_{\sigma }^{\dagger }{d}_{\sigma }+U{n}_{\downarrow }{n}_{\uparrow }$, where *ε* denotes the energy level and *U* stands for the repulsive interaction between opposite spin electrons. The QD is coupled to both external reservoirs via ${H}_{\beta -QD}={\sum }_{k,\sigma }\,({V}_{k\beta }{d}_{\sigma }^{\dagger }{c}_{k\sigma \beta }+h\mathrm{.}c.)$, where *V*_*kβ*_ denote the matrix elements. In a wide bandwidth limit, it is convenient to introduce the auxiliary couplings ${{\rm{\Gamma }}}_{{\rm{\beta }}}=2\pi {\sum }_{k}\,|{V}_{k\beta }{|}^{2}\,\delta (\omega -{\xi }_{k\beta })$, which can be assumed constant. It has been shown^57–60^, that for |*ω*| ≪ Δ the superconducting electrode induces the static pairing inside the quantum dot, ${H}_{S}+{H}_{S-QD}\approx -\,\frac{{\Gamma }_{S}}{2}({d}_{\uparrow }{d}_{\downarrow }+{d}_{\downarrow }^{\dagger }{d}_{\uparrow }^{\dagger })$. We make use of this low energy model, whose extension to arbitrary values of Δ has been discussed for instance in ref.^33^.

The zero-energy end modes of the topological nanowire can be modeled by the following term^47^

2 $${H}_{MQD}=i{\varepsilon }_{m}{\eta }_{1}{\eta }_{2}+\lambda ({d}_{\uparrow }^{\dagger }{\eta }_{1}+{\eta }_{1}{d}_{\uparrow })$$

with the hermitian operators ${\eta }_{i}={\eta }_{i}^{\dagger }$ and *ε*_*m*_ accounts for overlap between the Majorana quasiparticles. We recast these Majorana operators by the standard fermionic ones^5^ ${\eta }_{1}=\frac{1}{\sqrt{2}}(f+{f}^{\dagger })$ and ${\eta }_{2}=\frac{-i}{\sqrt{2}}(f-{f}^{\dagger })$ so that (2) can be expressed as

3 $${H}_{MQD}={t}_{m}({d}_{\uparrow }^{\dagger }-{d}_{\uparrow })(f+{f}^{\dagger })+{\varepsilon }_{m}{f}^{\dagger }f-\frac{{\varepsilon }_{m}}{2},$$

where $${t}_{m}=\lambda /\sqrt{2}$$.

### Spectrum of uncorrelated quantum dot

We first consider the uncorrelated QD case (*U* = 0). Let us calculate the retarded Green’s function ${\mathscr{G}}(\omega )=\langle \langle {\rm{\Psi }};{{\rm{\Psi }}}^{\dagger }\rangle \rangle$ defined in the matrix notation ${\rm{\Psi }}=({d}_{\uparrow },{d}_{\downarrow }^{\dagger },f,{f}^{\dagger })$

4 $$\mathop{\mathrm{lim}}\limits_{U=0}{{\mathscr{G}}}^{-1}(\omega )=(\begin{array}{cccc}\omega -\varepsilon +i{{\rm{\Gamma }}}_{N}\mathrm{/2} & {{\rm{\Gamma }}}_{S}\mathrm{/2} & -{t}_{m} & -{t}_{m}\\ {{\rm{\Gamma }}}_{S}\mathrm{/2} & \omega +\varepsilon +i{{\rm{\Gamma }}}_{N}\mathrm{/2} & 0 & 0\\ -{t}_{m} & 0 & \omega -{\varepsilon }_{m}-{t}_{m}^{2}/b & -{t}_{m}^{2}/b\\ -{t}_{m} & 0 & -{t}_{m}^{2}/b & \omega +{\varepsilon }_{m}-{t}_{m}^{2}/b\end{array}),$$

where *b* = *ω* + *ε* + *i*Γ_*N*_/2 − (Γ_*S*_/2)^2^/(*ω* − *ε* + *i*Γ_*N*_/2). For *ε*_*m*_ = 0 (in absence of any overlap between the Majorana modes) this Green’s function (4) simplifies to

5 $${{\mathscr{G}}}_{11}(\omega )=\frac{\omega +\varepsilon +i\frac{{{\rm{\Gamma }}}_{N}}{2}}{{D}_{1}(\omega )}+\frac{2{t}_{m}^{2}{(\omega +\varepsilon +i\frac{{{\rm{\Gamma }}}_{N}}{2})}^{2}}{D(\omega )},$$

6 $${{\mathscr{G}}}_{22}(\omega )=\frac{\omega -\varepsilon +i\frac{{{\rm{\Gamma }}}_{N}}{2}}{{D}_{1}(\omega )}+\frac{2{t}_{m}^{2}{(\frac{{{\rm{\Gamma }}}_{S}}{2})}^{2}}{D(\omega )},$$

7 $${{\mathscr{G}}}_{12}(\omega )=\frac{-\,\frac{{{\rm{\Gamma }}}_{S}}{2}}{{D}_{1}(\omega )}-\frac{2{t}_{m}^{2}(\omega +\varepsilon +i\frac{{{\rm{\Gamma }}}_{N}}{2})\frac{{{\rm{\Gamma }}}_{S}}{2}}{D(\omega )},$$

where $D(\omega )\equiv {D}_{1}(\omega )[\omega {D}_{1}(\omega )-4{t}_{m}^{2}(\omega +i{\Gamma }_{N}\mathrm{/2)}]$ and *D*_1_(*ω*) ≡ (*ω* + *i*Γ_*N*_/2)^2^ − *ε*^2^ − (Γ_*S*_/2)^2^. The first terms on r.h.s. of the Eqs (5–7) represent the Green’s function of QD coupled only to N and S electrodes (*t*_*m*_ = 0) and additional terms are contributed by the Majorana quasiparticles. In the superconducting atomic limit (Γ_*N*_ → 0) these Green’s functions are characterized by five poles: two of them corresponding to the Andreev bound states ($\pm \,\sqrt{{\varepsilon }^{2}+{({{\rm{\Gamma }}}_{S}/\mathrm{2)}}^{2}}$) and the other three states with energies (0, $\pm \sqrt{{\varepsilon }^{2}+{({{\rm{\Gamma }}}_{S}/\mathrm{2)}}^{2}+{\mathrm{(2}{t}_{m})}^{2}}$) resulting from the Majorana quasiparticles.

**Figure 2:**
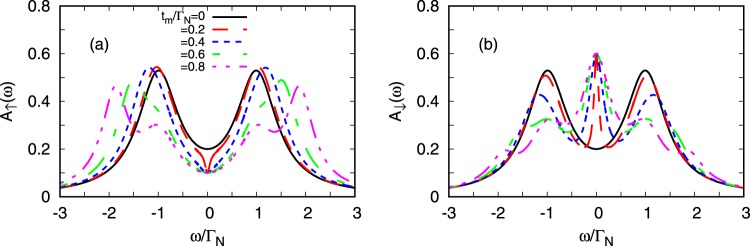
Free quasiparticle spectrum. The normalized spectral function ${A}_{\sigma }(\omega )=\frac{\pi }{2}{{\rm{\Gamma }}}_{N}{\rho }_{\sigma }(\omega )$ of the uncorrelated dot *U* = 0 obtained for Γ_*S*_ = 2Γ_*N*_, *ε* = 0 and various couplings *t*_*m*_.

Figure 2 shows the spin-resolved normalized spectral function $${A}_{\sigma }(\omega )=\frac{\pi }{2}{{\rm{\Gamma }}}_{N}{\rho }_{\sigma }(\omega )$$, where $${\rho }_{\sigma }(\omega )=-\,\frac{1}{\pi }$$$${\rm{Im}}\,{\langle \langle {d}_{\sigma };{d}_{\sigma }^{\dagger }\rangle \rangle }_{\omega +i{0}^{+}}$$, obtained for the uncorrelated QD at half-filling (*ε* = 0) for various couplings *t*_*m*_. As a reference shape, we display the spectrum in the absence of the Majorana quasiparticles (*t*_*m*_ = 0), revealing the Andreev quasiparticle peaks at $$\omega =\pm \,\sqrt{{\varepsilon }^{2}+{({{\rm{\Gamma }}}_{S}/\mathrm{2)}}^{2}}$$ whose broadening is described by Γ_*N*_. For *t*_*m*_ ≠ 0 the spin-resolved spectra are no longer identical due to the direct (indirect) coupling of ↑ (↓) QD electrons with the side-attached Majorana state. The most significant differences show up near *ω* ~ 0. In particular, direct hybridization of ↑ electrons depletes their spectrum near the Majorana state. Exactly at *ω* = 0 the spectral function is reduced by half, $${A}_{\uparrow }{\mathrm{(0)}}_{|{t}_{m}\ne 0}=0.5{A}_{\uparrow }{\mathrm{(0)}}_{|{t}_{m}=0}$$, similarly to what has been reported for the same geometry with both non-superconducting leads^44,50,56^. Contrary to this behavior, the spin-↓ electrons (indirectly coupled to the Majorana state via on-dot pairing) clearly gain the electronic states. Again, at *ω* = 0 the spectral function *A*_↓_(0) does not depend on *t*_*m*_ (unless *t*_*m*_ vanishes). This constructive feedback of the side-attached Majorana state on spin-↓ electrons has no analogy to any normal systems^44,50,56^. Upon increasing the coupling *t*_*m*_, we observe a gradual splitting of the Andreev quasiparticles, leading to the emergence of the effective *molecular* structure. We can notice some differences appearing in the spectrum *A*_*σ*_(*ω*) of spin-↑ and spin-↓ electrons, especially in the low energy region.

### Subgap Andreev transport

Low energy quasiparticles of quantum dot side-attached to the Majorana mode can be probed in our setup (Fig. 1) only indirectly, via the tunneling current. When voltage *V* applied between the normal tip and superconducting substrate is smaller than the energy gap Δ, at low temperatures the charge transport is solely due to the Andreev reflections^61^. For noninteracting systems such transport mechanism can be quantitatively determined from the Landauer-type formula

8 $${I}_{A}(V)=\frac{e}{h}\,\int \,d\omega \,{T}_{A}(\omega )[f(\omega \,-\,eV)-f(\omega \,+\,eV)],$$

where *f*(*x*) = [1 + exp(*x*/*k*_*B*_*T*)]^−1^ is the Fermi distribution. The energy-dependent transmittance

9 $${T}_{A}(\omega )={{\rm{\Gamma }}}_{N}^{2}\,{|{{\mathscr{G}}}_{12}(\omega )|}^{2}+{{\rm{\Gamma }}}_{N}^{2}\,{|{{\mathscr{G}}}_{21}(\omega )|}^{2}$$

describes a probability of electron (from STM tip) with spin *σ* to be converted into a hole (reflected back to the STM tip) with an opposite spin $\bar{\sigma }$, injecting one Cooper pair into the superconducting substrate. The same expression (8) is valid (but only approximately) for the correlated quantum dots^62^. The corresponding differential conductance *G*_*A*_(*V*) = *dI*_*A*_(*V*)/*dV* can detect the subgap quasiparticle states, even though the particle and hole degrees of freedom are mixed with each other^60^. In particular, at zero temperature the differential conductance simplifies to ${G}_{A}(V)=\frac{2{e}^{2}}{h}[{T}_{A}(\omega \,=\,+eV)+{T}_{A}(\omega \,=\,-\,eV)]$.

**Figure 3:**
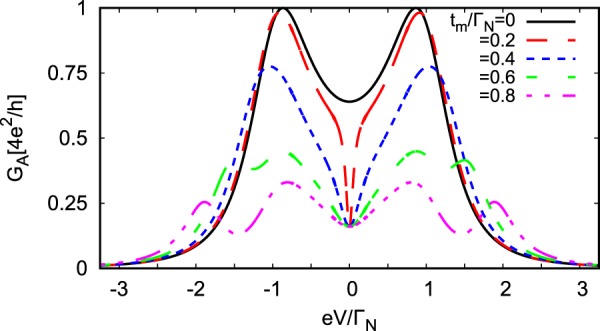
Subgap conductance. The differential Andreev conductance obtained for the same model parameters as in Fig. 2.

Figure 3 shows the differential Andreev conductance obtained at zero temperature for different values of *t*_*m*_, assuming *ε*_*m*_ = 0. We observe that for finite couplings *t*_*m*_ ≠ 0 the linear Andreev conductance *G*_*A*_(*V* = 0) drops to the value $\frac{1}{4}{G}_{A}{(V\mathrm{=}0)}_{{t}_{m}\mathrm{=0}}$. This result is qualitatively different from what has been obtained for N-QD-N junctions, where $G{(V=0)}_{{t}_{m}\ne 0}\,=\,\frac{3}{4}G{(V=0)}_{{t}_{m}=0}$^44^. Upon increasing the coupling *t*_*m*_ the nonlinear conductance *G*_*A*_(*V* ≠ 0) develops four local maxima, two of them at $\pm \sqrt{{\varepsilon }^{2}+{({\Gamma }_{S}\mathrm{/2)}}^{2}}$ and additional pair at $\pm \,\sqrt{{\varepsilon }^{2}+{({{\rm{\Gamma }}}_{S}/\mathrm{2)}}^{2}+{\mathrm{(2}{t}_{m})}^{2}}$. These local maxima are no longer equal to the perfect Andreev conductance 4*e*^2^/*h*. They originate from the Andreev states mixed with the Majorana quasiparticle (see Fig. 2).

In N-QD-N junctions with the side-attached Majorana nanowire the weak coupling *t*_*m*_ leads to the fractional Fano-type interference patterns^63^. In consequence, the density of states is reduced by half and the corresponding linear conductance drops to 3/4 of its original value, namely to ${e}^{2}/h+\frac{1}{2}{e}^{2}/h$ as compared to the maximum 2*e*^2^/*h* for *t*_*m*_ = 0 case. In our N-QD-S setup (Fig. 1) both spins participate in forming the local pairs, therefore Andreev current [dependent on the squared anomalous Green’s functions ${\mathscr{G}}$_12_(*ω*) and ${\mathscr{G}}$_21_(*ω*)] is characterized by the linear conductance *G*_*A*_(*V* = 0) reduced down to 25% for arbitrary coupling *t*_*m*_ ≠ 0 (Fig. 3).

### Majorana signatures in the correlated quantum dot

We now analyze the case of correlated quantum dot, focusing on the subgap Kondo effect originating from the Coulomb potential *U* and the coupling Γ_*N*_ to the normal STM tip. In the absence of the Majorana quasiparticle it has been shown^58,59^, that upon increasing the ratio Γ_*S*_/*U* the subgap Kondo peak gradually broadens^39,40^. This behavior occurs elusively when approaching the quantum phase transition from the spinful configuration side^57^. Our main purpose here is to examine how this subgap Kondo effect (appearing at zero energy) gets along with the leaking Majorana mode. Some earlier studies of the correlated quantum dot coupled to both normal (conducting) electrodes in presence of the side-attached Rashba chain indicated a competition between the Kondo and Majorana physics^44,50,51,56,64,65^. For sufficiently long wire (*ε*_*m*_ = 0) the Kondo effect is preserved only for the spin-↓ channel (which is not coupled to the Majorana zero-energy mode), whereas for the other spin-↑ channel there appears a dip in the spectral density at *ω* = 0 (reminiscent to what we observed in the upper panel of Fig. 2). In consequence, the total transmission is partly blocked, suppressing the linear conductance from 2*e*^2^/*h* to the fractional value 3*e*^2^/2*h*^43,44,50,51,56^. On the other hand, for short Rashba wires (*ε*_*m*_ ≠ 0), the Kondo peak survives in both spin channels, however, with its width affected by *ε*_*m*_. All initial Kondo features are fully recovered in both of the spin-channels only for *ε*_*m*_ $\gg$ (|*ε*|, *U*, Γ).

**Figure 4:**
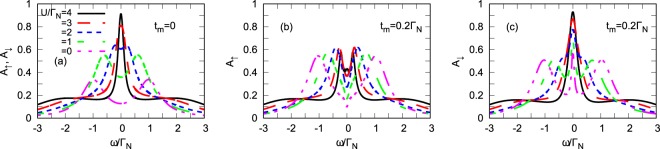
Spectrum of correlated dot. The spin-resolved spectral function *A*_*σ*_(*ω*) obtained by the SOPT method at zero temperature for the half-filled quantum dot (*ε* = −*U*/2), using *t*_*m*_ = 0 (left panel) and *t*_*m*_/Γ_*N*_ = 0.2 (middle/right panels).

When the correlated quantum dot is embedded between the metallic and superconducting leads (N-QD-S), the subgap Kondo effect is controlled by *U*/Γ_*S*_ ratio and *ε*^39,40,58,60,66^, which decide whether QD ground-state is the (spinful) doublet $|\sigma \rangle$ or the (spinless) BCS-type $u|0\rangle -v|\uparrow \downarrow \rangle$ configuration. In particular, for the half-filled QD $(\varepsilon =-\,\frac{U}{2})$ the BCS singlet is realized for *U* < Γ_*S*_, whereas the doublet is preferred for *U* > Γ_*S*_^57^. Obviously, the Kondo physics might occur only in the latter case, owing to antiferromagnetic exchange interactions driven between the QD and normal lead^40,67^. Figure 4 shows the corresponding spectral functions obtained at zero temperature by perturbative treatment of the Coulomb potential. The panel (a) refers to N-QD-S junction in the absence of the Majorana mode. In the weak interaction *U* regime, the spectral function is characterized by two Andreev peaks. When approaching *U* ≈ Γ_*S*_, these quasiparticle peaks merge, signaling a quantum phase transition (formally for Γ_*N*_ ≠ 0 it becomes a continuous crossover). In the strongly correlated limit (*U* > Γ_*S*_), we observe development of the subgap Kondo peak at *ω* = 0 whose width gradually shrinks upon increasing the ratio of *U*/Γ_*S*_^40,58^. In the presence of side-attached nanowire, the Majorana mode has completely different influence on each spin channel (panels b and c in Fig. 4). In some analogy to the non-interacting case the spectral function *A*_↑_(*ω*) is partly depleted near *ω* ~ 0 (due to destructive interference caused by the Majorana mode^37^), whereas the other spectral function *A*_↓_(*ω*) shows an opposite effect. For the latter case the Majorana mode contributes some electronic states near zero energy, therefore the Kondo peak becomes magnified.

**Figure 5:**
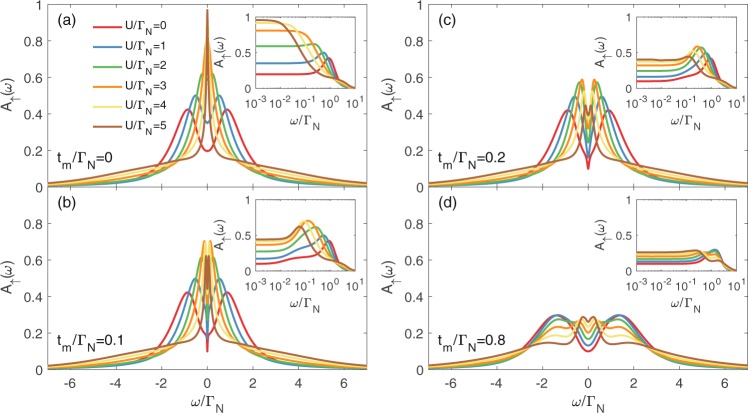
Spectrum of spin-↑ electrons. The normalized spectral function *A*_↑_(*ω*) for spin ↑ obtained from NRG calculations for Γ_*S*_ = 2Γ_*N*_, various ratios of *U*/Γ_*N*_ and several values of the coupling *t*_*m*_, as indicated. The other parameters are *ε* = −*U*/2, *ε*_*m*_ = 0 and Γ_*N*_ = *D*/50, with *D* the band halfwidth.

**Figure 6:**
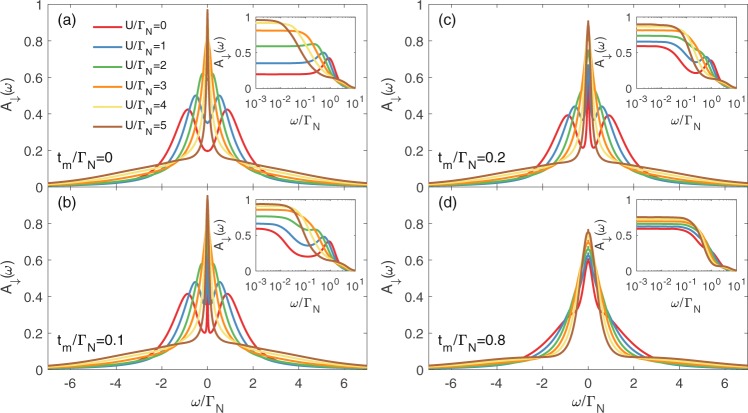
Spectrum of spin-↓ electrons. The spectral function *A*_↓_(*ω*) obtained by the NRG calculations for the same set of parameters as in Fig. 5.

Figures 5 and 6 present the spectral functions of spin-↑ and spin-↓ electrons obtained by the unbiased NRG calculations (see Methods for details). To inspect what happens to the Kondo state due to the side-attached Majorana mode, we display (in the insets) the low energy spectrum in the logarithmic scale. Quasiparticle states of spin-↑ electron (directly coupled to the Majorana mode) are strongly suppressed near *ω* ~ 0. In the weak Majorana-dot coupling regime (b & c panels) such effect originates from the destructive quantum interference^37^. However, for stronger couplings (e.g. for *t*_*m*_ = 0.8Γ_*N*_), QD electrons are substantially mixed with the Majorana mode and, in consequence, *A*_↑_(*ω*) develops novel (molecular) structure, revealing suppression of the zero-energy quasiparticles. This is particularly evident in the inset of Fig. 5(d). The spin-↓ sector (Fig. 6) reveals an opposite tendency. In this case, the Majorana mode indirectly affects the states predominantly in the vicinity of *ω* ~ 0. In the weak coupling limit the Kondo effect (existing for *U* ≥ Γ_*S*_) seems to be robust but its shape slightly broadens (see the insets of panels b & c). In the molecular regime (panel d) the electronic states cumulate near the zero energy, forming a single peak. We interpret it as an *indirect leakage of the Majorana quasiparticle driven by the on-dot pairing*. Numerical results obtained by the NRG calculations qualitatively agree with the selfconsistent perturbative treatment. In the weak coupling limit (small *t*_*m*_), both methods show detrimental influence of the Majorana mode on the subgap Kondo effect of ↑ spin and less severe (almost neutral) effect on ↓ spin sector. In the latter case the Kondo peak seems to be robust (it merely broadens). On the other hand, for the QD strongly coupled to the topological nanowire, we find that the Majorana mode strongly affects both spin sectors, substantially redistributing their quasiparticle spectra. Under such circumstances the Kondo state is hardly evident.

### Majorana and Kondo features in subgap transport

**Figure 7:**
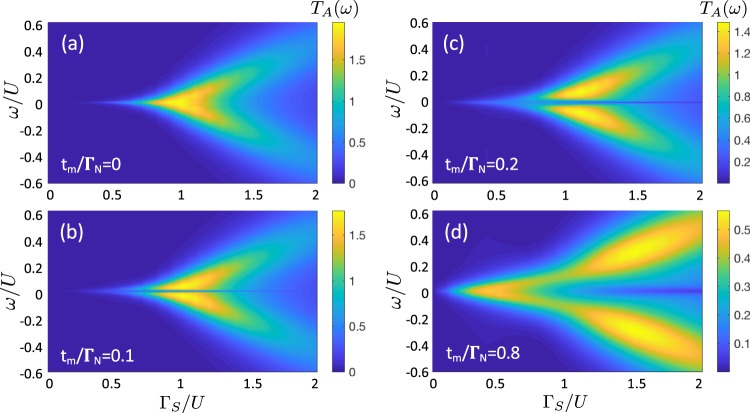
Kondo and Majorana signatures in subgap transmittance. The Andreev transmittance *T*_*A*_(*ω*) obtained by NRG for the half-filled QD and for different values of *t*_*m*_, as indicated. The other parameters are as in Fig. 5.

**Figure 8:**
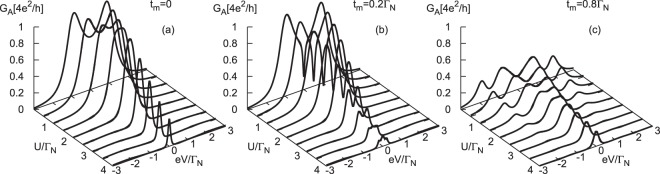
Nonlinear conductance of correlated dot. The differential subgap conductance *G*_*A*_(*V*) as a function of the applied voltage *V* and the Coulomb potential *U* obtained at *T* = 0 for the half-filled QD, using Γ_*S*_ = 2Γ_*N*_ and for different values of *t*_*m*_, as indicated.

Empirical detection of the subgap quasiparticles of correlated QD would be possible in our setup by the Andreev current conductance. The direct Andreev scattering, however, mixes the contributions of both spin channels to the effective transmittance (9). Figure 7 presents the transmittance *T*_*A*_(*ω*) obtained by NRG calculations for several couplings *t*_*m*_, as indicated. Variation of the differential Andreev conductance *G*_*A*_(*V*) with respect to the Coulomb potential *U* for the weak (b) and strong (c) coupling *t*_*m*_ limits is shown in Fig. 8. Nonequilibrium conditions have been taken into account within the perturbative approach, following the steps discussed by us in ref.^40^. In the weakly correlated case these plots resemble the results of the uncorrelated QD presented in Fig. 3. Remarkable changes appear in the strongly correlated limit, especially on the doublet side *U* ≥ Γ_*S*_.

To clarify the aforementioned behavior let us notice that in the absence of the Majorana mode (*t*_*m*_ = 0) the differential conductance is characterized by two peaks at bias *V*, coinciding with energies of the Andreev states. The zero-bias enhancement comes from the subgap Kondo effect, but solely in the doublet region (i.e. for *U* ≥ Γ_*S*_). Using the generalized Schrieffer-Wolff approach for the N-QD-S setup we have previously estimated^40^, that the effective Kondo temperature of the half-filled QD scales as *lnT*_*K*_ ∝ 1/[1 − (Γ_*S*_/*U*)^2^]. In particular, it yields enhancement of *T*_*K*_ with respect to Γ_*S*_ upon approaching the doublet-singlet transition. This unique behavior is valid for arbitrary Δ, as has been revealed by the NRG studies^39^. In the limit $U\gg {{\rm{\Gamma }}}_{S}$, the Andreev tunneling is strongly suppressed, because the off-diagonal Green’s function (characterizing efficiency of the induced on-dot pairing) nearly completely vanishes. These effects are illustrated in Fig. 8a.

The side-attached Majorana mode strongly affects the mentioned behavior. In the weak coupling limit (Fig. 8b) its influence is merely manifested near the zero-bias conductance. For ${{\rm{\Gamma }}}_{S} \sim U$, we observe a superposition of the leaking Majorana feature (whose width depends on *t*_*m*_) with leftovers of the Kondo peak, surviving only in the spin-↓ channel. For the strong *t*_*m*_-coupling case (Fig. 8c), the differential conductance *G*_*A*_(*V*) develops some novel molecular structure, characterized by four peaks. We interpret them as the bonding and anti-bonding mutations of the initial Andreev quasiparticles caused by strong hybridization with the Majorana mode. Upon increasing the Coulomb potential the internal peaks gradually merge into a single central one, whereas the external peaks loose their spectral weights.

### Majorana quasiparticles coupled to both spins

In realistic situations the spin-orbit coupling and the Zeeman effect break spin-rotational symmetry in Majorana nanowires. Spin is hence no longer a good quantum number. For this reason in proximitized nanowires with the strong spin-orbit interactions and in the presence of magnetic field effective intersite pairing is induced between the ‘tilted’ spins. Nevertheless, one can project this triplet pairing onto ↑ and ↓ components, estimating their amplitudes. Under these circumstances the Majorana quasiparticles emerge simultaneously in the spin-↑ and ↓ channels, but of course with different probabilities. The polarized Majorana quasiparticles have been indeed observed by A. Yazdani and coworkers in the STM measurement (using a ferromagnetic tip), for *Fe* atom nanochain deposited on superconducting Pb substrate^28^. Detailed analysis of this issue has been recently addressed by several groups^68–70^. To capture such magnetic polarization we generalize the initial model (3), assuming finite couplings of the Majorana modes to both spins of the quantum dot

10 $${H}_{MQD}=\sum _{\sigma }\,{t}_{m\sigma }\,({d}_{\sigma }^{\dagger }-{d}_{\sigma })(f+{f}^{\dagger })+{\varepsilon }_{m}{f}^{\dagger }f+\frac{{\varepsilon }_{m}}{2},$$

where *t*_*m*↑_ = *t*_*m*_*p* and *t*_*m*↓_ = *t*_*m*_(1 − *p*) with polarization *p* ∈ [0, 1]. The Green’s function of the uncorrelated QD is given by

11 $${{\mathscr{G}}}^{-1}(\omega )=(\begin{array}{cccccc}\omega -{\varepsilon }_{\uparrow }+i{{\rm{\Gamma }}}_{N}\mathrm{/2} & 0 & 0 & {{\rm{\Gamma }}}_{S}\mathrm{/2} & -{t}_{m\uparrow } & -{t}_{m\uparrow }\\ 0 & \omega +{\varepsilon }_{\uparrow }+i{{\rm{\Gamma }}}_{N}\mathrm{/2} & -{{\rm{\Gamma }}}_{S}\mathrm{/2} & 0 & {t}_{m\uparrow } & {t}_{m\uparrow }\\ 0 & -{{\rm{\Gamma }}}_{S}\mathrm{/2} & \omega -{\varepsilon }_{\downarrow }+i{{\rm{\Gamma }}}_{N}\mathrm{/2} & 0 & -{t}_{m\downarrow } & -{t}_{m\downarrow }\\ {{\rm{\Gamma }}}_{S}\mathrm{/2} & 0 & 0 & \omega +{\varepsilon }_{\downarrow }+i{{\rm{\Gamma }}}_{N}\mathrm{/2} & {t}_{m\downarrow } & {t}_{m\downarrow }\\ -{t}_{m\uparrow } & {t}_{m\uparrow } & -{t}_{m\downarrow } & {t}_{m\downarrow } & \omega -{\varepsilon }_{m} & 0\\ -{t}_{m\uparrow } & {t}_{m\uparrow } & -{t}_{m\downarrow } & {t}_{m\downarrow } & 0 & \omega +{\varepsilon }_{m}\end{array}).$$

**Figure 9:**
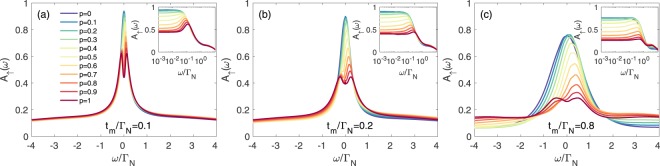
Spectrum for the polarized system. Spectral function *A*_↑_(*ω*) of the correlated QD obtained by NRG for varying *p*, using Γ_*S*_ = 2Γ_*N*_, *U* = 5Γ_*N*_ and several *t*_*m*_, as indicated. The other spectral function obeys the identity *A*_↓_(*ω*)_*p*_ = *A*_↑_(*ω*)_1−*p*_.

For reliable analysis of the correlation effects we have determined the spectral functions *A*_*σ*_(*ω*) by NRG calculations, focusing on the strong correlation limit *U* > Γ_*S*_. Figure 9 shows the spectral function *A*_↑_(*ω*) obtained in the Kondo regime for a number of polarizations *p* indicated in the legend. Spectra of the spin-up electrons for polarization *p* are identical with spectra of the spin-down electrons for polarization 1 − *p*, therefore, the behavior of *A*_↓_(*ω*) can be easily deduced from Fig. 9. We have inspected the spectral behavior in the weak (*t*_*m*_ = 0.1Γ_*N*_), moderate (*t*_*m*_ = 0.2Γ_*N*_) and strong coupling (*t*_*m*_ = 0.8Γ_*N*_) regions, respectively. We notice that polarization imposes a particle-hole asymmetry *A*_*σ*_(*ω*) ≠ *A*_*σ*_(−*ω*), both in the interferometric (small *t*_*m*_) as well as in the molecular (large *t*_*m*_) regime. Upon departing from the fully polarized case (*p* = 1 or *p* = 0) towards *p* → 0.5, the spin-selective influence gradually disappears. Nevertheless, for the realistic 30% polarization (*p* = 1 − 0.3) reported by Yazdani’s group^28^, we still clearly observe a destructive/constructive influence of the Majorana mode on the spectrum of spin up/down electrons.

Experimental observation of these spin-resolved and asymmetric features would be difficult by means of the local Andreev spectroscopy, because it mixes both spin degrees of freedom. For the same reason the local Andreev conductance would be rather weakly sensitive to polarization *p*. Another efficient tool for probing the magnetically polarized QD spectra might be nonlocal spectroscopy based on the selective equal spin Andreev reflection (SESAR) technique, briefly discussed in Methods. For some quantitative study one should apply the Bogoliubov de Gennes treatment for the tight binding description of the topological nanowires, which is however beyond the scope of the present study restricted to the low energy microscopic scenario.

## Discussion

We have analyzed the spin-resolved spectroscopic features of the quantum dot side-coupled to the topologically nontrivial superconducting nanowire, hosting the Majorana quasiparticles. Considering STM-type geometry, we have investigated the subgap electronic spectrum of QD and the Andreev conductance. In the uncorrelated case (*U* = 0), the Majorana quasiparticle induces either the zero-energy peak or dip in the QD spectrum, depending on its spin (Fig. 2). We assign it to the constructive or destructive quantum interference^63^. The direct Andreev conductance (equally sensitive to both spin sectors) would be predominantly affected by a destructive influence, manifested by the zero-bias dip in the weak hybridization *t*_*m*_ regime. In the molecular limit the QD spectrum and Andreev conductance are characterized by the emergent multi-peak structures.

We have also addressed the correlation effects, confronting them with the proximity induced on-dot pairing and the Majorana quasiparticle. Repulsive Coulomb interaction can cause the quantum phase transition from the spinless to spinful configuration^57,59^, qualitatively affecting the spin exchange mechanism (between QD and itinerant electrons of the normal electrode) leading to the subgap Kondo effect^39,40^. We have studied this mechanism in the presence of Majorana quasiparticles. Our calculations based on the selfconsistent perturbative treatment of the Coulomb potential *U* and using the unbiased NRG method reveal that the side-attached Majorana mode has spin-selective influence on the subgap Kondo effect. For spin-↑ electrons (directly coupled to the Majorana mode), it has a detrimental influence, whereas for the spin-↓ sector, the opposite tendency occurs. Such constructive/destructive influence of the Majorana mode on the proximitized QD could be probed either by spin-selective Andreev scattering^71^, spin-resolved current correlations^72^, or non-local spin blocking effect^73^.

Relationship between the Kondo state and the Majorana mode studied here differs from the previous considerations of the topological Kondo effect realized in the correlated nanowires^64,65,74–77^. In our context the subgap Kondo effect would be observable solely upon approaching the quantum phase transition of the correlated QD (manifested by the crossing Andreev quasiparticles). Since such crossing occurs at zero energy (i.e. the Fermi level), this effect should interfere with the zero-energy Majorana mode and the resulting would show up in the tunneling characteristics. Our study predicts the following features, which could be verified experimentally: (i) reduction of the linear conductance down to 25% of the perfect value typical for N-QD-S junctions^59^ in contrast to the reduction to 75% of the unitary value predicted for N-QD-N junctions^44^, (ii) suppression of the direct Andreev conductance near the parity changeover of the QD ground state (from the spinless to spinful configuration) in the weak hybridization (small *t*_*m*_) limit, (iii) development of the molecular structure in the strong hybridization (large *t*_*m*_) limit, in which the Majorana mode is combined with the Andreev and the subgap Kondo states, (iv) additional signatures of the Majorana mode appearing in the nonlocal Andreev scattering via the topological nanowire (see Methods), which could detect the singlet-doublet quantum phase transition.

## Methods

### Perturbative treatment of correlations

In a weakly interacting system the Coulomb term *Un*_↓_*n*_↑_ can be treated via perturbative scheme. It has been shown that the second-order perturbation theory (SOPT)^78^ properly accounts for essential features of the subgap Kondo effect^58^, at least qualitatively^40,67^. For the proximitized quantum dot one can formulate this SOPT approach, using the Dyson equation ${{\mathscr{G}}}^{-1}(\omega )={[{{\mathscr{G}}}^{U\mathrm{=0}}(\omega )]}^{-1}-{\rm{\Sigma }}\,(\omega )$ with the diagonal and off-diagonal parts of the matrix selfenergy^58^

12 $$\,{{\rm{\Sigma }}}_{11}(\omega )=U\langle {d}_{\downarrow }^{\dagger }{d}_{\downarrow }\rangle +{U}^{2}{\int }_{-{\rm{\infty }}}^{{\rm{\infty }}}\,\frac{(\,-\,\frac{1}{\pi })\,\,{\rm{I}}{\rm{m}}\,{{\rm{\Sigma }}}_{11}^{(2)}(\omega ^{\prime} )}{\omega -\omega ^{\prime} +i{0}^{+}}d\omega ^{\prime} ,$$

13 $$\,{\rm{\Sigma }}{}_{22}(\omega )=U\langle {d}_{\uparrow }{d}_{\uparrow }^{\dagger }\rangle +{U}^{2}\,{\int }_{-{\rm{\infty }}}^{{\rm{\infty }}}\,\frac{(\,-\,\frac{1}{\pi })\,\,{\rm{I}}{\rm{m}}\,{{\rm{\Sigma }}}_{22}^{(2)}(\omega ^{\prime} )}{\omega -\omega ^{\prime} +i{0}^{+}}d\omega ^{\prime} ,$$

14 $$\,{\rm{\Sigma }}{}_{12}(\omega )=U\langle {d}_{\downarrow }{d}_{\uparrow }\rangle -{U}^{2}\,{\int }_{-{\rm{\infty }}}^{{\rm{\infty }}}\,\frac{(\,-\,\frac{1}{\pi })\,\,{\rm{I}}{\rm{m}}\,\,{{\rm{\Sigma }}}_{12}^{(2)}\,(\omega ^{\prime} )}{\omega -\omega ^{\prime} +i{0}^{+}}d\omega ^{\prime} .$$

The terms proportional to *U* originate from the usual Hartree-Fock-Bogoliubov (static) approximation, whereas the second-order (dynamic) contributions $\,{{\rm{\Sigma }}}_{ij}^{(2)}(\omega )$ can be expressed by the following convolutions^67^

15 $$-\,\frac{1}{\pi }\,\,{\rm{I}}{\rm{m}}\,{{\rm{\Sigma }}}_{11(22)}^{(2)}\,(\omega )={\int }_{-{\rm{\infty }}}^{{\rm{\infty }}}\,[{{\rm{\Pi }}}_{1}(\omega +\omega ^{\prime} ){\rho }_{22(11)}^{+}(\omega ^{\prime} )+{{\rm{\Pi }}}_{2}\,(\omega +\omega ^{\prime} ){\rho }_{22(11)}^{-}(\omega ^{\prime} )]\,d\omega ^{\prime} ,$$

16 $$-\,\frac{1}{\pi }\,{\rm{I}}{\rm{m}}\,\,{{\rm{\Sigma }}}_{12}^{(2)}\,(\omega )={\int }_{-{\rm{\infty }}}^{{\rm{\infty }}}\,[{{\rm{\Pi }}}_{1}\,(\omega +\omega ^{\prime} ){\rho }_{21}^{+}\,(\omega ^{\prime} )+{{\rm{\Pi }}}_{2}\,(\omega +\omega ^{\prime} ){\rho }_{21}^{-}\,(\omega ^{\prime} )]\,d\omega ^{\prime} $$

with

17 $${{\rm{\Pi }}}_{\mathrm{1(2)}}\,(\omega )={\int }_{-\infty }^{\infty }\,[{\rho }_{11}^{-(+)}\,(\omega ^{\prime} ){\rho }_{22}^{-(+)}\,(\omega -\omega ^{\prime} )-{\rho }_{12}^{-(+)}(\omega ^{\prime} ){\rho }_{21}^{-(+)}(\omega -\omega ^{\prime} )]\,d\omega ^{\prime} .$$

The auxiliary functions ${\rho }_{ij}^{\pm }(\omega )\equiv \frac{-\,1}{\pi }{\rm{Im}}\,{{\mathscr{G}}}_{ij}^{0}(\omega \,+\,i{0}^{+})f(\,\pm \,\omega )$ are computed, using the uncorrelated Green’s functions (4) but with the Hartree-Fock-Bogoliubov (static) shifts taken into account^58^. We have selfconsistently determined the selfenergies (12–14) for sufficiently dense mesh of the discretized energy *ω*, slightly above the real axis.

### NRG calculations

The most reliable analysis of the interplay of correlation effects with electron-pairing and the leaking Majorana quasiparticle is possible within the numerical renormalization group (NRG) approach^79^. We have performed such calculations, focusing on the low energy (subgap) physics of the effective model

18 $$H={H}_{N}+{H}_{N-QD}+{H}_{QD}^{prox}+{H}_{MQD},$$

where *H*_*MQD*_ is defined in Eq. (3) and ${H}_{QD}^{prox}={H}_{S}+{H}_{S-QD}+{H}_{QD}\simeq {\sum }_{\sigma }\,\varepsilon {d}_{\sigma }^{\dagger }{d}_{\sigma }+U{n}_{\downarrow }{n}_{\uparrow }-\frac{{{\rm{\Gamma }}}_{S}}{2}\,({d}_{\uparrow }{d}_{\downarrow }+{d}_{\downarrow }^{\dagger }{d}_{\uparrow }^{\dagger })$ describes the interacting quantum dot with the proximity induced on-dot pairing. In practice, we have numerically investigated the following Hamiltonian

19 $$\begin{array}{c}H=\sum _{\sigma }\,\varepsilon {d}_{\sigma }^{\dagger }{d}_{\sigma }+U{n}_{\downarrow }{n}_{\uparrow }-\frac{{{\rm{\Gamma }}}_{S}}{2}\,({d}_{\uparrow }{d}_{\downarrow }+{d}_{\downarrow }^{\dagger }{d}_{\uparrow }^{\dagger })+{t}_{m}({d}_{\uparrow }^{\dagger }-{d}_{\uparrow })(f+{f}^{\dagger })+{\varepsilon }_{m}\,{f}^{\dagger }f-\frac{{\varepsilon }_{m}}{2}\\ \,+\sqrt{\frac{{{\rm{\Gamma }}}_{N}}{2\pi \rho }\,}\sum _{\sigma }\,[{d}_{\sigma }^{\dagger }{c}_{0\sigma N}+{c}_{0\sigma N}^{\dagger }{d}_{\sigma }]+\sum _{\sigma ,j\ge 0}\,{t}_{j}[{c}_{j\sigma N}^{\dagger }{c}_{j+1\sigma N}+{c}_{j+1\sigma N}^{\dagger }{c}_{j\sigma N}]\end{array}$$

where ${c}_{j\sigma N}^{(\dagger )}$ represent j-th site operators of the Wilson’s semi-infinite chain, *t*_*j*_ are the hopping integrals between the neighboring sites and we have assumed the flat density of states $\rho =\frac{1}{2D}$ of the normal lead with a cutoff $D\gg U$. This single-channel model (19) allowed for a good quality computational analysis. We have performed numerical calculations, using the Budapest Flexible DM-NRG code^80^ for constructing the zero-temperature density matrix of the system and calculating the spin-resolved spectral functions for arbitrary model parameters *U*, *ε*, Γ_*β*_, *t*_*m*_ and *ε*_*m*_. Since the coupling *t*_*m*_ to Majorana mode and superconducting pairing correlations Γ_*S*_ break the spin and charge symmetries, only the charge parity symmetry of the total Hamiltonian was used. In calculations we kept at least 1024 states per iteration and imposed the discretization parameter Λ = 2. Our results were averaged over *N*_*z*_ = 4 interleaved discretization^81^, using the logarithmic Gaussian broadening to obtain the smooth spectral functions. In this paper we focused on the half-filled case *ε* = −*U*/2, assuming *ε*_*m*_ = 0.

### Nonlocal Andreev transport

Let us finally discuss some additional contribution to the charge transport between *N* and *S* electrodes indirectly via the topological nanowire. In the subgap regime (where any single particle tunneling is prohibited) there might occur the nonlocal Andreev tunneling. Physically, it could originate from a particle-hole scattering through the anomalous terms

20 $${\langle \langle {d}_{\sigma };f\rangle \rangle }_{\omega +i{0}^{+}}\mathrm{.}$$

These propagators describe the indirect conversion of electrons (arriving from the metallic lead) to holes (injected to the topological nanowire) and further transmitting the pairs into the superconducting electrode. Such mechanism is analogous to the crossed Andreev reflections observed in three-terminal (or multiple) junctions. Let us notice, that the nonlocal Andreev mechanism can be realized individually for both spin orientations (although with significantly different probabilities). In particular, the nonlocal transfer of the spin-↑ channel would correspond to, so called, *selective equal spin Andreev reflection* (SESAR) proposed in ref.^71^. Such SESAR mechanism is allowed, because electrons of the topological nanowire are effectively bound into intersite pairs of the identical spins (i.e. triplet pairing)^19^. The spin-selective nonlocal Andreev transport has been shown to be a very useful tool for probing the spatial extent and magnetic polarization of the Majorana quasiparicles^68^.

**Figure 10:**
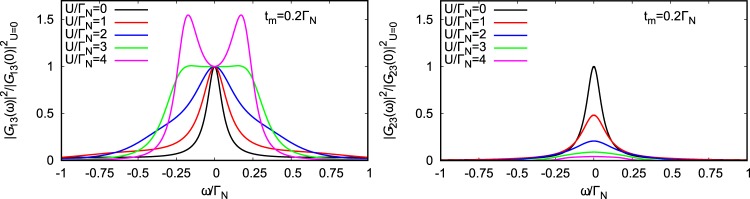
Efficiency of nonlocal transport. Energy dependence of the equal spin Andreev scattering obtained for various Coulomb potentials (indicated in the legend) and for *t*_*m*_ = 0.2Γ_*N*_, Γ_*S*_ = 2Γ_*N*_.

Within the low energy scenario (2) we can estimate only quantitatively such nonlocal Andreev scattering, by exploring the propagators (20). A quantitative analysis of the nonlocal Andreev conductance would require some microscopic description of the Rashba or helically ordered proximitized nanowire, using the Bogolubov de Gennes approach. Here we restrict to evaluation of the squared absolute values of terms (20), analogous to the direct Andreev transmittance (9). Figure 10 shows the results obtained within the selfconsistent perturbative treatment for the half-filled QD and for various *U*, as indicated. These spin-dependent quantities are normalized to their zero-energy values of the noninteracting case

$$\mathop{\mathrm{lim}}\limits_{U\to 0}{|{\langle \langle {d}_{\sigma };f\rangle \rangle }_{\omega +i{0}^{+}}|}^{2}=\frac{1}{16{t}_{m}^{2}}\times \{\begin{array}{ll}1 & \,\,{\rm{for}}\,\sigma =\uparrow ,\\ {({{\rm{\Gamma }}}_{S}{/{\rm{\Gamma }}}_{N})}^{2} & \,\,{\rm{for}}\,\sigma =\downarrow .\end{array}$$

We clearly notice that correlations lead to the completely different behavior in each spin component. The nonlocal (SESAR) Andreev transport of spin-↑ electrons is enhanced upon increasing the ratio *U*/Γ_*N*_. In the weak correlation limit *U *≤ Γ_*S*_ (when the QD ground state is in BCS-type configuration) it is characterized by a single peak at zero energy. In the strongly correlated case *U* ≥ Γ_*S*_ (corresponding to the spinful QD configuration) the nonlocal Andreev probability develops two-peak structure. Contrary to this behavior, the nonlocal Andreev transport of spin-↓ electrons is monotonously suppressed by the Coulomb potential. We assign such effect to the fact, that influence of the Majorana quasiparticle on spin-↓ electrons occurs indirectly via the particle-hole mixing. For strong enough Coulomb potential the on-dot pairing diminishes, therefore the nonlocal Andreev scattering of spin-↓ electrons is substantially suppressed. On the other hand, this parity changeover (from the spinless BCS-type to the spinful doublet configuration) is accompanied by evolution of the nonlocal (SESAR) Andreev scattering of spin-↑ electrons from a single to double peak behavior, which should be detectable by spin-resolved spectroscopic techniques^28,31^.
